# Assessing Pathogenicity for Novel Mutation/Sequence Variants: The Value of Healthy Older Individuals

**DOI:** 10.1007/s12017-012-8186-x

**Published:** 2012-06-16

**Authors:** Mayana Zatz, Rita de Cassia M. Pavanello, Naila Cristina V. Lourenço, Antonia Cerqueira, Monize Lazar, Mariz Vainzof

**Affiliations:** Departamento de Genética e Biologia Evolutiva, Centro de Estudos do Genoma Humano, Universidade de São Paulo, Rua do Matão, 106, Cidade Universitária, São Paulo, SP 05508-090 Brazil

**Keywords:** Genome sequencing, Unknown mutations, Dystrophin gene, Older relatives DNA

## Abstract

Improvement in DNA technology is increasingly revealing unexpected/unknown mutations in healthy persons and generating anxiety due to their still unknown health consequences. We report a 44-year-old healthy father of a 10-year-old daughter with bilateral coloboma and hearing loss, but without muscle weakness, in whom a whole-genome CGH revealed a deletion of exons 38–44 in the dystrophin gene. This mutation was inherited from her asymptomatic father, who was further clinically and molecularly evaluated for prognosis and genetic counseling (GC). This deletion was never identified by us in 982 Duchenne/Becker patients. To assess whether the present case represents a rare case of non-penetrance, and aiming to obtain more information for prognosis and GC, we suggested that healthy older relatives submit their DNA for analysis, to which several complied. Mutation analysis revealed that his mother, brother, and 56-year-old maternal uncle also carry the 38–44 deletion, suggesting it an unlikely cause of muscle weakness. Genome sequencing will disclose mutations and variants whose health impact are still unknown, raising important problems in interpreting results, defining prognosis, and discussing GC. We suggest that, in addition to family history, keeping the DNA of older relatives could be very informative, in particular for those interested in having their genome sequenced.

## Introduction

The reduced cost of DNA sequencing has launched several genome population projects in an attempt to clarify the contribution of genetic diversity to normal human as well as disease-related traits. Next-generation DNA sequencing can provide insight into different types of genetic variation that characterize the human genome, such as single-nucleotide polymorphisms, copy-number variation, mobile elements as well as the burden of deleterious variants that may be present in our genome (MacArthur et al. 2012; Quintana-Murci 2012). Several ongoing whole genome–sequencing projects are also focusing on centenarians or older individuals, in particular, to enhance our understanding on genetic versus environment contribution to healthy aging (Altshuler et al. 2010). Additionally, sequencing the genome of healthy older individuals will be extremely important as a database to interpret the significance of novel variants found in younger subjects, some of them associated with well-established Mendelian disorders as reported here.

Duchenne (DMD) and Becker (BMD) muscular dystrophies are X-linked allelic disorders caused by mutations in the dystrophin gene, which may result in the absence (DMD) or a defective (BMD) muscle protein dystrophin (Hoffman et al. 1987; Monaco et al. 1988). In DMD, which affects around 1 in 3,000/4,000 male births, the disease progression is very similar in all affected patients. Without any medical intervention, affected patients are usually confined to a wheelchair around age 10–12, and in the second decade, they are completely dependent for all activities. Death usually occurs as a result of respiratory failure or cardiac impairment in the second decade. Differently from DMD, BMD is characterized by a wide clinical variability. Some patients are confined to a wheelchair before age 20, while others may remain ambulant until late in life. Previous genotype:phenotype correlation studies have shown that the severity of the clinical course depends on the amount of muscle dystrophin and on the site of the mutation (Hoffman et al. 1988; Koenig et al. 1989; Vainzof et al. 1993). Deletions in the rod domain of the dystrophin gene are usually associated with a milder phenotype (Passos-Bueno et al. 1994), while those that involve the N or C-terminal domains cause a more severe course (Hoffman et al. 1988), although prognosis in younger patients can be difficult.

## Results

**Fig. 1:**
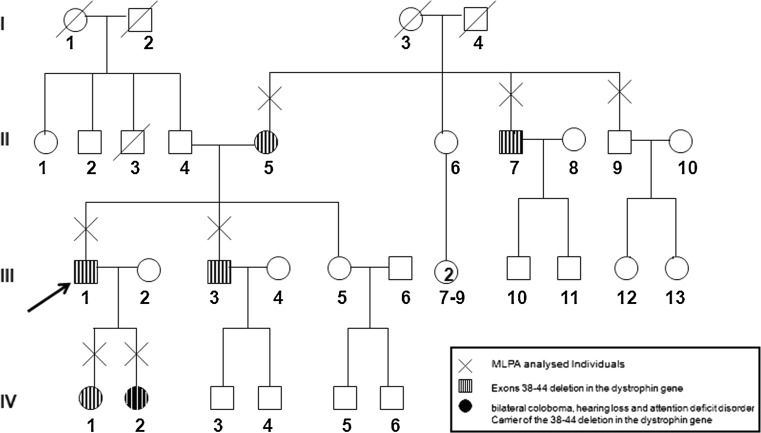
Family pedigree

Here, we report an apparently clinically normal man carrying a deletion in the dystrophin gene who was indirectly ascertained through his daughter (IV-2, Fig. 1). This 10-year-old girl was referred for genetic studies due to facial dysmorphism, neurological and behavioral abnormalities, and attention deficit disorder with mild cognitive impairment, but no muscle weakness. Whole-genome array CGH performed elsewhere (Genome Dx Report) revealed her to carry a 179-kb deletion in the dystrophin gene that is apparently unrelated to her condition. This deletion was inherited from her father who was referred to us for further evaluation and genetic counseling.

The father, a 44-year-old healthy male, was clinically and molecularly evaluated in the Human Genome Research Center at the University of São Paulo. All studies were done following written informed consent.

Clinical and neurological examination showed that this father, who was our proband, is completely asymptomatic. He has no visible calf hypertrophy and is able to run and jump. He reportedly plays soccer every weekend and has no signs of fatigue, cramps, or other symptoms.

Pedigree analysis (Fig. 1) revealed that our proband (III-1) has two daughters (IV-1 and IV-2, aged, respectively, 16 and 10 years) and two younger siblings: a brother (III-3, aged 39) and a sister (III-5, aged 43), both clinically normal. His father (II-4, currently 70 years old) had two brothers (II-2 and II-3) and one sister (II-1). One of the paternal uncles (II-3) died of heart attack at age 39, and both the father and older paternal uncle have a heart condition. His mother (II-5), who is currently 64, has three younger siblings: one sister (II-6) who is 61, and two brothers (II-7 and II-9) aged, respectively, 56 and 44 years old. None of them have any clinical condition.

Complementary exams on the father showed that his serum creatine kinase was borderline (223 U/l; normal, up to 189 U/l) (Zatz et al. 1978). DNA analysis through MLPA (multiplex ligation-dependent probe amplification) screening for whole dystrophin gene confirmed the presence of an “in frame” deletion spanning exons 38 to 44.

**Fig. 2:**
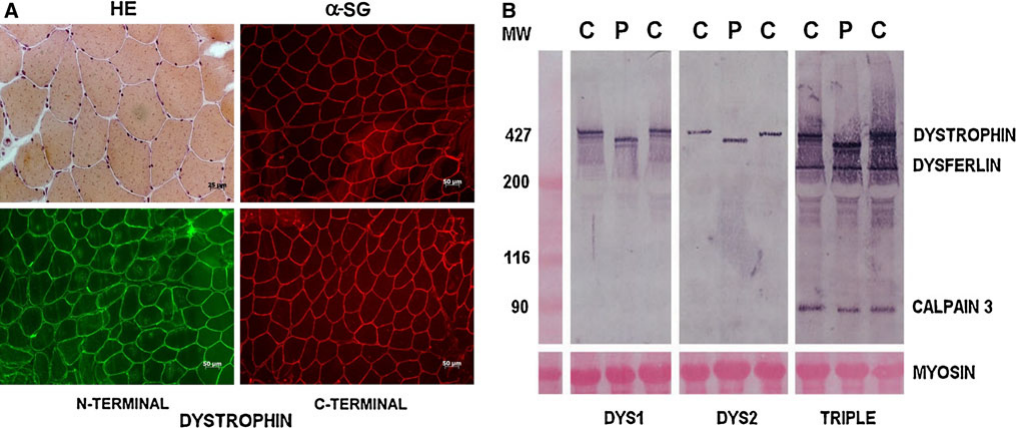
Histological (HE) and immunohistochemical analysis of the proband’s muscle biopsy, showing in **a**—normal muscle histology in HE staining, and normal dystrophin (antibodies against the N-terminal and C-terminal domains) and sarcoglycan (α-SG) distribution in muscle sarcolemma. In **b**—Western blot analysis for dystrophin showing a dystrophin band of ~390 kDa, in normal quantity, using antibodies against the rod domain (DYS1) and C-terminal domain (DYS2). In the triple reaction, calpain-3 and dysferlin bands are normal

Muscle biopsy revealed histological characteristic of normal muscle (Fig. 2a). Immunofluorescence muscle protein analysis for dystrophin, using antibodies against the N-terminal and C-terminal regions of the protein, showed a normal and continuous sarcolemmal pattern of distribution (Fig. 2a). Sarcoglycans proteins were also normally distributed (Fig. 2a). Through Western blot analysis, a strong dystrophin band of ~390 kDa was observed with antibodies against the rod domain (DYS1) and C-terminal domain (DYS2), compatible with the size expected for the transcript of a gene with the partial deletion of exons 38–44. Calpain-3 and dysferlin bands were normal (Fig. 2b).

## Discussion

Improvement in DNA technology is increasingly identifying unexpected mutations in healthy persons, causing anxiety particularly when no information is available about their possible health consequences. “In frame” deletions of variable extent, mostly in the rod domain of the dystrophin gene, have been reported before in individuals with no or with very mild symptoms, such as elevated serum CK or myalgia (Ferreiro et al. 2009; Gospe et al. 1989; Ishigaki et al. 1996; Melis et al. 1998).

Two separate mutation prediction databases have classified the molecular defect found in our proband as compatible with Becker muscular dystrophy (The UMD-DMD France mutation database) or of unknown consequence (http://www.umd.be/DMD/4DACTION/Web_Large_rearrangement/c.5326_6438de). On the other hand, a duplication involving the same exons was reported in a Chinese patient with a DMD phenotype (http://www.dmd.nl/#eupdate, and Yuge et al. 1999). This mutation has never been found among 1,600 DMD/BMD patients analyzed in our center, 982 of them through MLPA technology (unpublished data). Therefore, it was difficult to conclude whether the lack of symptoms of our proband represents an exception or whether he could be reassured about his prognosis. In addition to his own future, this information was also important for genetic counseling of his daughters since it is expected that both carry the 38–44 deletion and thus have a chance of 50 % of transmitting this mutation to their male offspring.

In the present case, we did not know whether the 38–44 dystrophin deletion was a de novo mutation in the father or was already segregating in the family. This possibility was discussed during genetic counseling, and it was explained that DNA analysis of older family members could be informative in addressing this question. Although at first our proband was reluctant at the possibility of causing anxiety in other healthy relatives, he decided to contact them and several key members agreed to have their DNA screened for mutations in the dystrophin gene.

MLPA analysis in 4 additional relatives (III-3, II-5, II-7, and II-9) revealed that three of them carry the same 38–44 deletion: his mother (II-5), his younger brother (III-3), and his maternal uncle (II-7). Both grandparents are deceased, and therefore, we could not investigate further the origin of this mutation, but according to our proband′s information, they had no muscle weakness. It is not possible to rule out that our proband will have some muscle pathology later in life or that the same mutation could be pathogenic in an individual with a different genetic background. However, the finding of the same mutation in his asymptomatic brother and uncle who is currently 56 and also completely asymptomatic suggests that this mutation is unlikely to result in muscle weakness, at least in the present family.

Advances in genome-sequencing analysis will uncover genetic mutations and variants whose impacts in health are still unknown. This will raise important problems in interpreting results and defining prognosis as well as in genetic counseling of at-risk family members. Indeed, the recent analysis of 185 genomes has shown that all humans carry many genetic variants predicted to cause loss of function of protein-coding genes and approximately 20 genes are completely inactivated (MacArthur et al. 2012).

The finding of unexpected and novel mutations will probably be ever more frequent and their impact will have to be analyzed carefully during genetic counseling. Many disease-causing mutations reported in the literature have later been shown to be benign polymorphisms or only partially penetrant (Altshuler et al. 2010). Other publications on individual genome sequences have also found homozygosity for alleged severe disease mutations despite no evidence for the associated phenotype in the sequenced individual (Lupski et al. 2010). In short, the available mutation databases are quite imperfect guides to assess sequence variant pathogenicity. On this respect, deep genome analysis of healthy octogenarians (currently underway in our center), the whole-genome sequencing of 1,000 healthy older individuals (the Scripps Wellderly Study), and of 100 centenarians may bring valuable information, in particular for adult onset disorders.

In the family presented here, the observation that the same mutation was present in older healthy relatives was very helpful and reassuring, in particular because the penetrance of a specific mutation can vary on different genetic backgrounds. Therefore, in addition to population studies, we suggest keeping the DNA of your older family members. It could be very informative, in particular for those who want to have their own genome sequenced.
